# Apparent Lack of Benefit of Combining Repetitive Transcranial Magnetic Stimulation with Internet-Delivered Cognitive Behavior Therapy for the Treatment of Resistant Depression: Patient-Centered Randomized Controlled Pilot Trial

**DOI:** 10.3390/brainsci13020293

**Published:** 2023-02-09

**Authors:** Medard Kofi Adu, Reham Shalaby, Ejemai Eboreime, Adegboyega Sapara, Mobolaji A. Lawal, Corina Chew, Shelley Daubert, Liana Urichuck, Shireen Surood, Daniel Li, Mark Snaterse, Mike Mach, Pierre Chue, Andrew J. Greenshaw, Vincent I. O. Agyapong

**Affiliations:** 1 Department of Psychiatry, Faculty of Medicine, Dalhousie University, Halifax, NS B3H 4R2, Canada; 2 Department of Psychiatry, Faculty of Medicine and Dentistry, University of Alberta, 8440 112 St NW, Edmonton, AB T6G 2B7, Canada; 3 Alberta Health Services, Addiction, and Mental Health, Edmonton, AB T5J 3E4, Canada

**Keywords:** repetitive transcranial magnetic stimulation, treatment-resistant depression, internet-based cognitive behavioral therapy, MoodGYM, major depressive disorder

## Abstract

**Background:** Treatment-resistant depression (TRD) is considered one of the major clinical challenges in the field of psychiatry. An estimated 44% of patients with major depressive disorder (MDD) do not respond to two consecutive antidepressant therapies, and 33% do not respond to up to four antidepressants. Over 15% of all patients with MDD remain refractory to any treatment intervention. rTMS is considered a treatment option for patients with TRD. Likewise, iCBT is evidence-based, symptom-focused psychotherapy recommended for the treatment of TRD. **Objective:** This study aimed to evaluate the initial comparative clinical effectiveness of rTMS treatment with and without iCBT as an innovative intervention for the treatment of participants diagnosed with TRD. **Methods:** This study is a prospective two-arm randomized controlled trial. Overall, 78 participants diagnosed with TRD were randomized to one of two treatment interventions: rTMS sessions alone and rTMS sessions plus iCBT. Participants in each group were made to complete evaluation measures at baseline, and 6 weeks (discharge) from treatment. The primary outcome measure was baseline to six weeks change in mean score for the 17-item Hamilton depression rating scale (HAMD-17). Secondary outcomes included mean baseline to six-week changes in the Columbia suicide severity rating scale (CSSRS) for the rate of suicidal ideations, the QIDS-SR16 for subjective depression, and the EQ-5D-5L to assess the quality of health in participants. **Results:** A majority of the participants were females 50 (64.1%), aged ≥ 40 39 (50.0%), and had college/university education 54 (73.0%). After adjusting for the baseline scores, the study failed to find a significant difference in the changes in mean scores for participants from baseline to six weeks between the two interventions under study on the HAMD-17 scale: F (1, 53) = 0.15, *p* = 0.70, partial eta squared = 0.003, CSSRS; F (1, 56) = 0.04 *p* = 0.85, partial eta squared = 0.001, QIDS-SR16 scale; F (1, 53) = 0.04 *p* = 0.61, partial eta squared = 0.005, and EQ-5D-VAS; F (1, 51) = 0.46 *p* = 0.50, and partial eta squared = 0.009. However, there was a significant reduction in means scores at week six compared to baseline scores for the combined study population on the HAMD-17 scale (42%), CSSRS (41%), QIDS-SR16 scale (35%), and EQ-VAS scale (62%). **Conclusion:** This study did not find that combined treatment of TRD with rTMS + iCBT (unguided) was superior to treatment with rTMS alone. Our findings do not support the use of combined treatment of rTMS + iCBT for the management of TRD disorders.

## 1. Introduction

Treatment-resistant depression (TRD) is a major clinical feature of patients treated for major depressive disorder (MDD). TRD is considered one of the major clinical challenges in the psychiatry community. It is estimated that 44% of MDD patients do not respond to two consecutive antidepressant therapies, and 33% do not respond to four antidepressants [1]. Despite the high TRD frequency, it seems that the concept is poorly understood [2]. Perhaps TRD may be a multidimensional disorder which includes several subtypes with different neurobiological bases [3,4]. Thus, a clear consensus for a standard definition for TRD is missing from the literature [5], leading to many misdiagnoses and inadequacies in the treatment of patients who are considered to be resistant to treatment in MDD [6]. Although researchers have used a variety of criteria to define TRD [7], a common agreement is that a person with MDD is “resistant” based on no adequate response to at least two trials of antidepressants from different pharmacological classes despite a well-managed treatment protocol [8]. The determination of resistance is only confirmed after the patient has been evaluated to ascertain the accuracy of diagnosis, adequate dosing, treatment adherence, and whether the worsening of the patient’s condition is influenced by other confounding factors, such as coexisting medical or psychiatric disorders [9].

The prognosis of TRD seems bleak, as it is characterized by a profound worsening of the quality of life, a greater rate of mortality, decreased productivity, more hospitalizations, higher individual and community-related healthcare costs, and higher rates of suicidal ideation [10,11,12]. It is estimated that about 30% of TRD patients attempt suicide at least once in their lifetime [12,13], twice the rate of suicide attempts in non-treatment-resistant MDD patients (estimated between 8.4% [14] and 15.9% [15]) and about 15 times higher than the rate estimated for the entire European population [14,16]. Considering the high suicide risk of TRD patients, it is of utmost importance to evaluate whether specific treatments might impact the rate of suicidal ideation in this cohort of patients. Despite the rapid growth of the variety of treatment choices for TRD, the condition represents a domain of unmet therapeutic need. There are few psychopharmacological agents approved for the management of TRD, and overall treatment outcomes remain poor [17]. Regarding antidepressant treatments for TRD, their efficacy is comparable between classes. Thus, the choice of any particular antidepressant medication is determined by the evaluation of side effects, history of treatment response in the patient and relatives, and, to some extent, the cost of medication [9].

Amid uncertainty in the management of TRD due to the limited evidence-based optimal pharmacologic and psychotherapeutic interventions for TRD [18,19], repetitive transcranial magnetic stimulation (rTMS) has been considered an essential investigational treatment technique that is fit for this purpose [20]. Several randomized controlled studies on rTMS have focused on TRD patients; however, most of these investigated effects in the treatment of a combination of drug-resistant patients and did not strictly evaluate the effectiveness of rTMS in patients with TRD [21].

rTMS is a non-invasive focal brain stimulation that is considered to be an essential technique in neuropsychiatry treatment due to its ability to produce direct effects on a range of measures of brain function [22,23]. rTMS is extensively used in the management of TRD and has been greatly studied as a major technique in many other psychiatric disorders; thus, it is deemed a brain-system-based neuromodulation treatment based on its focus on the direct target of the neural circuitry of disorders [24]. High-frequency (≥1 Hz) and low-frequency (≤1 Hz) methods are the two major forms of rTMS techniques applied in clinical practice. While high-frequency rTMS is believed to produce a highly stimulating effect on the cerebral cortex, low-frequency rTMS is thought to produce an inhibitory effect [25,26]. More importantly, at a time when researchers are struggling to find a much better treatment for resistant depression, rTMS has earned itself a place in the management of depressive disorders globally, and more research is being conducted to evaluate the same. Study findings indicate asymmetry in the functioning of patients diagnosed with MDD [27]. Therefore, researchers have to apply an inhibitory rTMS stimulation (low frequency) to the right dorsolateral prefrontal cortex (DLPFC) and excitatory stimulation (high frequency) to the left DLPFC in TRD patients [28].

Cognitive behavioral therapy (CBT) is another treatment option that has a proven empirical base for the management of TRD [29]. However, CBT comes with a major challenge of access and dissemination, and this is partly due to the insufficient number of trained therapists [30]. Digital technology interventions are emphasized by experts in the field of psychiatry as a major means to transform the delivery of healthcare [31,32]. There has been a sharp recent increase in the use of web-based technologies in support of the application of cognitive behavioral therapy (CBT) [33,34], and studies have been conducted in this regard to demonstrate the efficacy of internet-based CBT (iCBT) in TRD [35]. The literature on therapist-supported iCBT trials for depression indicates a significant and stable clinical effect on MDD [36]. iCBT interventions, administered with or without therapist assistance, are typically referred to as guided and unguided iCBT, respectively. Unguided iCBT is considered more affordable and more accessible than guided iCBT, but previous results indicate that therapist assistance generally leads to better outcomes [37]. iCBT uses web-based software programs to deliver these interventions [38]. Interactions on these software platforms are provided as assessment instruments and treatment materials delivered in the form of videos, audio, and text messages [39].

Several programs for iCBT are identified in the literature for the management of depression, including the UK-developed cognitive-behavioral Beating the Blues (BTB) [40]. The BTB consists of eight person-centered online cognitive modules on depression which takes up to 50 min to complete [40,41]. MoodGYM is a popular internet-based iCBT platform that was developed in Australia, consisting of an interactive self-guided book that leads a patient to learn and practice skills that help to prevent and manage symptoms of depression and anxiety. MoodGYM comprises five unique modules targeted at the relationship between thoughts and emotions, identifying cognitive distortions and negative thoughts, techniques to adjust negative thoughts, assertiveness and self-esteem training, behavioral activation, and problem-solving [42]. MoodGYM helps in identifying negative thoughts and teaches practical strategies for managing the negative thoughts and beliefs to reduce the dysfunctional thinking of MDD patients. Several studies have attested to the efficacy of MoodGYM for MDD for both outpatients and inpatients in clinical settings [33,43,44,45,46,47]. According to the World Health Organization (WHO), it is an international priority to increase the coverage of interventions and evidence-based treatments for TRD globally [48]. In addition, rTMS is considered a treatment option for patients with TRD who are refractory to antidepressant treatment, while iCBT is an evidence-based, symptom-focused psychotherapy recommended for the treatment of TRD. It is not currently known if adding unguided iCBT will enhance patients’ responses to rTMS treatments. This project was designed to evaluate the initial comparative clinical effectiveness of rTMS treatments with and without unguided iCBT in TRD patients as an alternative to the currently known pharmacological and other treatment options.

## 2. Methods

### 2.1. Study Design

This study is a two-arm parallel design and rater-blinded randomized controlled pilot trial. The study was conducted at the Addiction and Mental Health Clinic, 108th Street Building, and at the Alberta Day Hospital’s rTMS clinic in Edmonton, Alberta. Participants were recruited and randomized into one of two treatment interventions under study (rTMS alone and rTMS plus unguided iCBT) to receive active treatment for six weeks. Assessment measures were conducted at baseline and six weeks (discharge) for all participants.

### 2.2. Institutional Review Board Approval

The study protocol [49] received ethical clearance from the Health Ethics Research Board of the University of Alberta (Pro00094208) and was registered with clinicaltrials.gov: (registration number: NCT04239651; pre-result). The study was conducted per the Declaration of Helsinki (Hong Kong Amendment) [50] and Good Clinical Practice (Canadian Guidelines). Written informed consent was obtained from each study participant.

### 2.3. Inclusion and Exclusion Criteria

To be eligible for the study, participants must have a diagnosis of TRD [6] based on the Diagnostic and Statistical Manual of Mental Disorders (DSM) 5 criteria and have failed two or more standard antidepressant treatments during the current episode, having a Hamilton Depression Rating Scale (17-HAM-D) score of 10 or more and met the general criteria for receipt of publicly funded rTMS treatment in the Edmonton locations, between 18 and 65 years, with a good understanding of the English language, with access to a computer with internet, have fair computer/internet skills (thus, having the ability to navigate the MoodGYM program on a computer with ease), and be able and willing to provide informed consent.

The exclusion criterion was diagnosis with the following conditions (current unless otherwise stated):

1. A neurological disorder, including a history of seizures, cerebrovascular disease, primary or secondary tumors in the central nervous system, stroke, cerebral aneurysm, movement disorder, or any lifetime history of loss of consciousness due to head injury.
2. Any current personality disorder that would interfere with participation in the study or might affect cognition and ability to participate meaningfully, as well as mental retardation identified through medical history or by the investigator.
3. A current amnestic disorder, dementia, or delirium, as defined by a Montreal Cognitive Assessment score of ≤16, or any other neurological or mental disease that might affect cognition or the ability to participate in CBT meaningfully.
4. Participation in any drug or device clinical trial in the six weeks (42 days) prior to the screening visit and/or participation in another clinical trial for the duration of the study.
5. Participants who are pregnant/breastfeeding.
6. Discovery and/or the sudden appearance of any condition or circumstance from the above list that, in the opinion of the investigator, has the potential to prevent study completion and/or to have a confounding effect on outcome assessments.

Determination of eligibility for the rTMS treatment was assessed by the rTMS-trained psychiatrists (M.L. and D.L.) at the study sites. Patients not meeting all of the inclusion criteria were excluded from the study.

### 2.4. Recruitment Procedures

After a patient was evaluated and found to be eligible for rTMS treatment, a research team member (MA) introduced the study to him/her with the aid of an information leaflet that contained brief details about the study protocol, answering any questions he/she had before aiding him/her in signing a consent form. The process of recruitment and obtaining consent form was completed within the rTMS eligibility assessment week, a week before the initial rTMS administration to the participant. Once recruited, the patient was assigned a study identification number—passed to an independent statistician for randomized group allocation. Participants in both arms of the study were educated on the protocol for the study. The participants were informed of the routine activities that take place during each visit to the rTMS clinic. As part of their participation, participants were pre-informed about the completion of standard questionnaires at baseline and six weeks (discharge).

### 2.5. Randomization and Blinding

The participants were block randomized into either the rTMS alone group or the rTMS + iCBT group, with randomization codes secured on a password-protected computer. Primary outcome assessors were blinded to treatment-group allocation by not involving them in discussions about study participants and not granting them access to the secured database which contained the randomization codes.

### 2.6. Intervention

All participants were scheduled to receive 30 sessions of unilateral rTMS over six weeks. The intervention was provided face-to-face and on an individual basis. The study followed the rTMS protocol, as predetermined by the Alberta Health Services Strategic Clinical Network for Addiction and Mental Health. The motor threshold (MT) assessment is essential in that it aids in the selection of the required stimulation intensity for each patient for inclusion in the treatment. MT assessment is a measure of the minimum intensity of TMS output needed to elicit a motor response in the participating TRD patients in at least 50% of all attempts. Thus, low-frequency (1 Hz) inhibitory right dorsolateral prefrontal cortex (R-DLPFC) at 120% MT, 20 trains (60 pulses/train at 60 s), was used. Total pulse of 1200 for 20 min and high frequency (10 Hz) excitatory stimulation left dorsolateral prefrontal cortex (L-DLPFC) at 120% MT, 75 trains with an intertrain interval of 11 s. Total pulses of 3000 for 19 min. The Magventure rTMS machine was used. The coil type was the figure of 8, and the angle between the coil and the head was 45 degrees. The cortex area was targeted by using the 5 cm technique. The prefrontal cortex stimulation site was determined as 5 cm anterior further ahead of the motor strip in the parasagittal line. The management of missed sessions was ensured by contacting the patient to ensure adherence to the treatment. The patients were continuously monitored, and an attending nurse +/− the psychiatrist remained with the patient during the whole session, checking on any expected side effect. Attending at least 25 out of 30 sessions was considered a complete treatment.

In addition to the 30 sessions of rTMS, participants recruited to the combined treatment intervention group (rTMS + iCBT) were guided to enroll in the iCBT program (MoodGYM). The MoodGYM program comprises 5 unique modules that are targeted as the relationship between thoughts and emotions, identifying cognitive distortions and negative thoughts, techniques to adjust negative thoughts, assertiveness and self-esteem training, behavioral activation, and problem-solving. With the aid of their unique login information, participants in this arm of the study were enabled to participate in 12 one-hour sessions of iCBT over six weeks. The MoodGYM sessions were scheduled at least twice a week, at three days intervals (Preferably Tuesdays and Thursdays). Participants completed a 1 h session of iCBT in the comfort of their homes a night before the rTMS session or an hour before the rTMS session on the same day. Participants within the rTMS + iCBT arm of the study were sent reminders via text messaging on the days and times of their 1-h sessions of iCBT and also encouraged to assess the MoodGYM program even outside the scheduled periods, at their convenience.

### 2.7. Sample Size Calculation

Consistent with the idea that this is a pilot study, with no established effect size data available to assist in power and sample size calculations, the researchers used data from participants who could be enrolled within the existing operational resources. This method is acceptable for pilot studies involving novel interventions and has been described by Haynes et al. as using “the participants I can get” [51]. In this way, the study was limited to a sample size of 80, with 40 participants to be recruited for each arm.

### 2.8. Data Collection

Social demographics, including gender, age, and educational level attained, and clinical characteristic information were routinely collected for all patients at baseline and six weeks for patients receiving rTMS at the two sites. The sociodemographic variables and clinical variables were collected at pre-rTMS treatment (baseline) and post-rTMS treatment (six weeks), using the 17-item Hamilton Depression Scale (HAMD) [52], which is used to quantify depression symptom severity in patients diagnosed with MDD; the Columbia Suicide Severity Rating Scale (CSSRS), which is used to screen and evaluate a person’s level of suicidal ideations [53]; the self-reported 16 items Quick Inventory Depression Scale (QIDS-SR16), which is used to evaluate the nine diagnostic symptoms domain of the DSM-IV [54]; and the EQ-5D-5L, which is used to assess the quality of overall health status [55]. The HAMD has strong psychometric properties [56], including internal reliability evaluated by Cronbach’s alpha statistic [57] of ≥0.70, which is deemed sufficient reliability [58]. Ratings on the HAMD are determined on a semi-structured clinical interview, producing the highest score of 52. A total of 8 of the 17 items are rated on a 5-point scale of 0–4 (0 = absent, 1 = doubtful or mild, 2 = mild to moderate, 3 = moderate to severe, and 4 = very severe), and the remaining 9 on a 3-point scale of 0–2 (0 = absent, 1 = doubtful or mild, and 2 = clearly present). The ratings are based on the individual rater’s clinical judgment; both severity and frequency of the symptoms are taken into account [59]. A total score of 0–7 is considered to be being normal, 8–16 suggests mild depression, 17–23 suggests moderate depression, and scores over 24 are indicative of severe depression, with the maximum score being 52 on the 17-point scale [60].

The screen version of CSSRS is made up of 6 questions. Users are tasked to respond “Yes” or “No” to whether they have thought about suicide, have acted or plan to act, or whether they attempted suicide or plan to attempt suicide. Each of the 6 questions evaluates a different component of the respondent’s suicide ideation severity and behavior. This measuring tool is scored as low, moderate, or high risk, depending on positive answers (yes) to the various questions. Once a respondent answers in the affirmative (yes) to Question 2, he/she is instructed to respond to Questions 3–5. If the respondent answers “no” to Question 2, he/she may skip to Question 6. Responding “yes” to any of the 6 items may imply a need for referral to a mental healthcare professional, and responding “yes” to Question 4, 5, or 6 indicates high risk. This scale has a wide evidence base and is supported by SAMHSA, the CDC, the FDA, the NIH, the WHO, and many other credible institutions. There is a good internal consistency for the CSSRS intensity of ideation subscale with Cronbach’s alpha values ranging between 0.73 and 0.93 [53]. The questions on the scale include (1) wish to be dead, (2) non-specific suicidal thoughts, (3–5) more specific suicidal thoughts and intent to act, and (6) suicidal behavior over the respondent’s lifetime and past 3 months.

QIDS-SR16 has an internal scale consistency of (coefficient α = 0.86) [61] and is a valid depression screening instrument for patients in different age categories [62,63]. Three domains (sleep, appetite/weight, and restlessness/agitation) are scored based on the highest score obtained on two or more questions. The remaining domains are each scored on a single item. All items are scored from 0 to 3, and greater scores reflect severe psychopathology. The total scores on this scale range from 0 to 27. A score of ≤5 indicates no depression, 6 to 10 represents mild depression, 11 to 15 indicates moderate depression, 16 to 20 indicates severe depression, and a total score greater than 21 reflects a very severe depression [64].

The EQ-5D-5L consists of the EQ-5D descriptive system and the EQ visual analog scale. The descriptive system is made up of five domains: mobility, self-care, usual activities, pain/discomfort, and anxiety/depression [55]. Each of the dimensions consists of 5 levels (no problems, slight problems, moderate problems, severe problems, and extreme problems). The score from the five dimensions, when combined, indicates the quality of health of the patient. The patient’s self-rated quality of health is recorded on a vertical visual analog scale (EQ-VAS) from 0 to 100, with 100 representing the best health you can imagine and 0 representing the worst health you can imagine. This can be used as a quantitative measure of health outcomes that reflect the patient’s judgment [55,65]. The EQ-5D-5L has adequate psychometric properties in patients with major depression. The reliability of EQ-5D-5L per calculation using Cronbach’s alpha coefficient was 0.77, which is superior to the minimum acceptable value of 0.70. Regarding convergent and discriminant validity, there are high correlations between the PHQ-9 and the EQ-5D-5L index and the anxiety/depression dimension (−0.52 and 0.56, respectively). For known-groups validity, patients with a greater level of depression and those with poorer general health have significantly lower scores on the EQ-5D-5L (*p* < 0.001) [66].

### 2.9. Primary Outcome Measure

The primary outcome measure was the mean change in the HAMD-17 from baseline to six weeks for the intervention and control groups.

### 2.10. Secondary Outcome Measures

Secondary outcomes included changes in mean scores from baseline to six weeks on the CSSRS, the QIDS-SR16, and the EQ-VAS for the intervention and control groups. Other secondary outcomes included differences in the prevalence of the clinical conditions measured by the HAMD, CSSRS, the QIDS-SR16, and the EQ-5D-5L between the two interventions (rTMS alone and rTMS + iCBT) at discharge (six weeks).

### 2.11. Exploratory Outcomes

An exploratory outcome was the overall change in mean scores on the HAMD-17, the CSSRS, the QIDS-SR16, and the EQ-VAS from baseline to six weeks for all participants in the study.

### 2.12. Statistical Analysis

We completed data analysis, following an intention-to-treat basis with (39) patients in the rTMS alone group and (39) patients in the rTMS plus iCBT group. Data were analyzed using the statistical package for social sciences SPSS version 25 (IBM Corporation, 2011) [57]. Descriptive data for baseline parameters were presented using frequencies and percentages among the two intervention groups and compared by Chi-Square/Fischer Exact tests for categorical variables and the independent sample *t*-test for continuous variables. Differences in the effectiveness of the two interventions were assessed using one-way between-groups analysis of covariance (ANCOVA) analysis, comparing the changes in mean scores from baseline to six weeks on the HAMD-17, CSSRS, QIDS-SR16, and EQ-VAS scales between the two interventions groups, while controlling for their respective baseline scores. Four models were run for each outcome scale. The independent variables consisted of the type of intervention (rTMS alone and rTMS plus iCBT), while the scores on the HAM-D-17, CSSRS, QID-SR16, or EQ-VAS scales at 6 weeks were considered the dependent variables for each of the analyses. Baseline scores on the respective scales were used as the covariates in the analyses. Preliminary checks were conducted to ensure there was no violation of the assumptions of normality, linearity, homogeneity of variance, homogeneity of regression slopes, or reliable measurement of the variate. For participants with missing six weeks data, the last observation (baseline/interim measures) was imputed before performing sensitivity analyses of covariance to explore the impact of imputation of data loss on HAMD-17, CSSRS, QIDS-SR16, and EQ-VAS scores at six weeks.

Chi-square/Fischer Exact tests were utilized to compare the prevalence of clinician-rated MDD, using the HAMD scale of two categories with a cutoff score of 17; suicidal ideations, using the CSSRS scale of two categories; patient self-rated MDD, using the QIDS-SR16 scale of two categories; and quality of health, using the EQ-5D-5L five subscales, between the two intervention groups at six weeks, and also for the overall sample, between prevalence at baseline and six weeks for each variable.

Chi-squared/Fisher’s Exact tests and, where necessary, also post hoc analysis, were used to compare categorical scores on the EQ-5D-5L scale related to mobility, self-care, usual activities, pain/discomfort, and anxiety/depression for the two groups We reported corresponding z-scores, adjusted residuals, and *p*-values.

Additionally, a paired *t*-test was used to examine the changes in baseline and six-week mean scores on the HAMD, CSSRS, QIDS-SR16, and EQ-VAS for participants who completed the instruments at both time points.

Frequencies and percentages were used for reporting categorical variables, while mean scores, confidence intervals, and effect sizes were used when reporting on continuous variables. There was no imputation for missing data, and the total numbers reported represent the total responses recorded for each variable. The two-tailed α-level criterion for statistical significance was set at *p* ≤ 0.05.

## 3. Results

**Figure 1:**
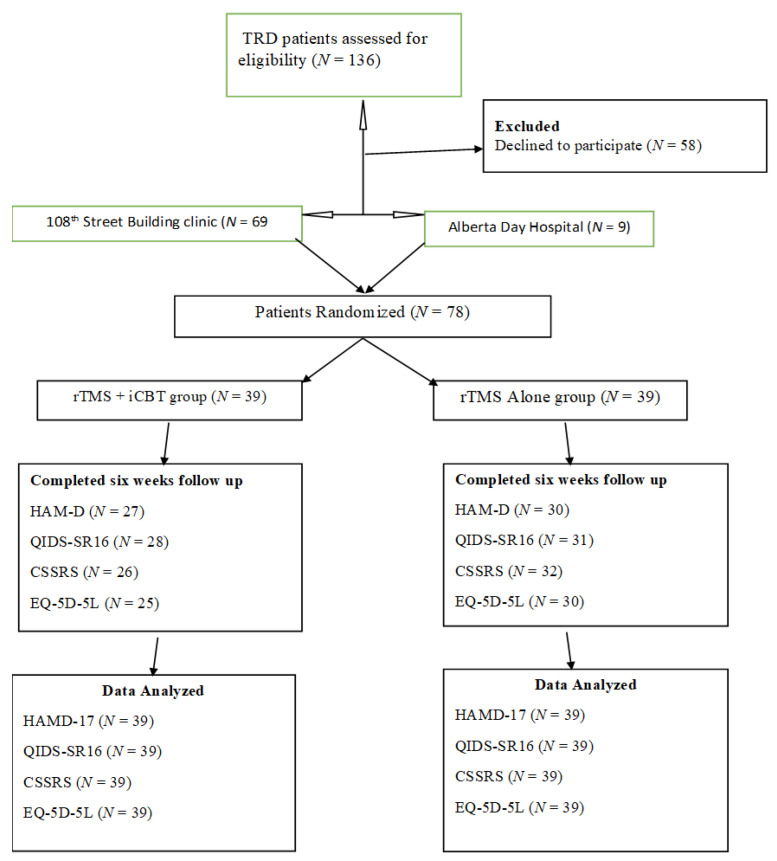
Study flow chart.

The research team approached 136 patients who had been assessed and met the criteria to receive rTMS treatment. Out of this number approached for eligibility for the study, 58 individuals declined the invitation to take part in the study and cited reasons such as (“Not interested in research studies” and “Time factor”). The study realized a total of 78 participants randomized, with 39 recruited into each of the two treatment interventions (rTMS alone and rTMS with iCBT). Figure 1 shows the study flowchart. The overall effectiveness of any treatment intervention must acknowledge both its efficacy and any safety and tolerability factors. In this regard, rTMS treatment appeared to be reasonably well-tolerated by the study’s participants. However, the most common side effects witnessed and reported were transient headaches, dizziness, and scalp discomfort at the stimulation site. These mild side effects disappeared during treatment or soon after treatment.

Table 1 provides the baseline distribution of sociodemographic and clinical characteristics of the two study groups. The data presented in Table 1 suggest that a majority of the participants were females 50 (64.1%), aged ≥ 40 39 (50.0%), and had college/university education 54 (73.0%).

Regarding the clinical characteristics at baseline, the prevalence of clinician-rated likely MDD was 36 (51.4%), and by contrast, patient-rated likely MDD was 64 (90.1%) and the presence of suicidal ideation was 52 (71.2%). There were no statistically significant differences between the two intervention groups at baseline for mean scores on the HAMD, the CSSRS, the QIDS-SR16, and the EQ-VAS.

### 3.1. Primary Outcome Measures

To compare the effectiveness of the treatment interventions (rTMS alone and rTMS plus iCBT) for the management of TRD amongst participants, ANCOVA was conducted as shown in Table 2. Regarding the HAMD scale, after controlling for the baseline scores, the results revealed no significant differences between the two treatment intervention groups at six weeks (F (1, 53) = 0.15, *p* = 0.70, partial eta squared = 0.003). After data imputation, there remains no significant difference in the mean changes in HAMD scores from baseline to six weeks between the intervention and control groups (*p* > 0.05). There was a relationship between the baseline intervention score and six weeks’ intervention score on the HAM-D scale, as evidenced by the partial eta-squared value of 0.11.

### 3.2. Secondary Outcome Measures

An ANCOVA analysis was performed on data gathered from the CSSRS scale to assess differences in the change mean scores from baseline to six weeks in suicidal ideation between the study groups (rTMS alone and rTMS plus iCBT). After controlling for baseline scores, we found that there were no significant differences between the two study groups at six weeks on the CSSR scale (F (1, 56) = 0.04 *p* = 0.85, partial eta squared = 0.001), as shown in Table 2. There was a relationship between the baseline intervention score and six weeks’ intervention score on the CSSRS scale, as evidenced by a partial eta-squared value of 0.36.

An ANCOVA was also performed on data from the QIDS-SR16 scale to assess differences in the change mean scores from baseline to six weeks for self-rated depression between the study groups (rTMS alone and rTMS plus iCBT). After we controlled for baseline scores, the analysis revealed no significant differences between groups at six weeks on the QIDS-SR16 scale (F (1, 53) = 0.04, *p* = 0.61, partial eta squared = 0.005), as shown in Table 2. There was a relationship between the baseline intervention score and six weeks’ intervention score on the QIDS-16 scale, as evinced by a partial eta-squared value of 0.28. The ANCOVA analysis of EQ-VAS data was conducted to assess differences in the change mean scores from baseline to six weeks for the quality of health between the two study groups. After we controlled for baseline scores, the results indicated no significant difference between groups at six weeks on the EQ-VAS (F (1, 51) = 0.46, *p* = 0.50, partial eta squared = 0.009), as displayed in Table 2.

After data imputation, there remains no significant difference in the mean changes in CSSRS, QIDS-SR16, and EQ-VAS scores from baseline to six weeks between the intervention and control groups (*p* > 0.05).

Table 3 illustrates the differences in the prevalence of the clinical conditions between the two interventions (rTMS alone and rTMS + iCBT) at discharge (six weeks). Overall, there was no significant difference between the two intervention groups on all measured scales. The Chi-square/Fischer exact values ranged from 0.51 to 2.95, while the *p*-values ranged from 0.20 to 0.91.

### 3.3. Exploratory Outcomes

Table 4 demonstrates the changes in study measures from baseline to discharge (six weeks) for all the study participants who completed both the baseline and six-week scales. The data in Table 4 suggest that the mean scores of the HAMD-17 at baseline (M = 16.25; SD = 5.29) and six weeks (M = 9.45; SD = 6.44), with t (68) = 7.46 (*p* = 0.001); CSSRS at baseline (M = 1.69; SD = 1.59) and six weeks (M = 1.00; SD = 1.27), with t (71) = 4.06 (*p* = 0.001); QIDS-SR16 at baseline (M = 16.79; SD =4.45) and six weeks (M = 10.88, SD = 5.22), with t (69) = 9.45 (*p* = 0.001); and EQ-VAS at baseline (M = 47.67; SD = 18.45) and six weeks (M = 62.56; SD = 19.75), with t (69) = 6.31 (*p* = 0.001), scales were significantly lower at 6 weeks compared to the baseline mean scores. This indicates an overall improvement in the severity of depressive symptoms, suicidal ideations, subjective depression symptoms, and the quality of health, regardless of the type of intervention. The effect size, as measured by Cohen’s d, for HAM-D-17, CSSRS, QIDS-SR16, and EQ-5D-5L was 1.15, 0.48, 1.23, and 0.78, respectively.

From Table 4, our results revealed a significant reduction in the mean score of all measured scales after week six compared to the baseline scores (HAM-D (42%), CSSRS (41%), QIDS-SR16 (35%), and EQ-VAS (62%))

Similar to the previous results, the data of Table 5 indicate statistically significant reductions in the prevalence of depression in participants after the six-week assessment when compared to the baseline assessment with MDD (33.9%), suicidal ideation (18.7%), and subjective depression (41.1%). Regarding the EQ-5D-5L scale, there were four subscales, namely mobility, self-care, usual activity, and pain/discomfort, that did not show a statistically significant association between their baseline and discharge values (*p* = 0.194. *p* = 0.252, *p* = 0.221, and *p* = 0.315, respectively). However, for the Anxiety/Depression Subscale (Table 5), the post hoc analysis using adjusted residuals indicated that baseline proportions (20%) of the respondents who reported being “Extremely anxious or depressed” were significantly reduced at six weeks (discharge) (3.6%) (z = 2, *p* = 0.046).

## 4. Discussion

### 4.1. Principal Findings

This study failed to establish a significant difference between outcomes for the two interventions under study (rTMS alone and rTMS plus iCBT) regarding the improvement of MDD symptoms, suicidal ideations, subjective depression symptoms, and the quality of health on all scales. However, in the exploratory analysis, there was a significant improvement in all sample participants regarding depressive symptoms, suicidal ideations, subjective depression, and the quality of health from baseline after introducing the intervention for six weeks.

In the wake of a global search for better, safer, and cost-effective management of TRD, many treatment alternatives to traditional psychopharmacology have been introduced into the therapeutic space of psychiatric healthcare, including rTMS and CBT. There have been many studies conducted concerning the actual efficacy of these interventions in TRD separately [20,67,68,69,70]. Though results from the various studies seem to support the efficacy of these interventions, data concerning their combined efficacy are lacking in the literature. To the best of our knowledge, the concomitant application of internet-delivered psychotherapy (iCBT) with rTMS has not been studied before and this study is the first study of its kind. Therefore, the results generated in this study provide a concrete basis for further evaluating the practical application and efficacy of using a novel combination of these two treatment modalities (rTMS plus iCBT) with a larger sample size and target population of different characteristics.

Our study yielded some interesting outcomes regarding the changes in the clinician, as well as the self-evaluated severity and prevalence of depression, suicidal ideations, and the quality of health in the TRD patients under study. Overall, there was a significant improvement observed at 6 weeks (discharge) in participants’ mean scores on HAM-D-17 (41.8%), CSSRS (40.8%), QIDS-SR16 (35.2%), and EQ-5D-5L (61.56) from the baseline scores. This suggests that, overall, the treatment intervention was effective in reducing the depressive symptoms and suicidal symptoms and improving the quality of health in the TRD patients with high-to-moderate effect sizes (1.15, 0.48, 1.23, and 0.78, respectively). With regard to prevalence, our results demonstrated statistically significant reductions in the prevalence of major depressive disorder, as well as the suicidal symptoms, and an improvement in the quality of health in all participants at the week-six assessment compared to the baseline assessment (33.9%, 18.7%, and 41.1%, respectively).

While there were significant improvements in the symptoms of depression and suicidal ideations and an improvement in the quality of health of all participants at week six, this study failed to demonstrate a significant difference between the two interventions (rTMS alone and rTMS plus iCBT). Thus, after controlling for the baseline scores, the results revealed no significant differences between the two treatment intervention groups at six weeks on the 17-item HAM-D (*p* = 0.70), the CSSR scale (*p* = 0.85, QIDS-SR16 (*p* = 0.61), and EQ-5D-5L (*p* = 0.50). This implies that there is no added value for adding iCBT as an adjunctive treatment with rTMS for the treatment of TRD.

The present findings should, of course, be interpreted cautiously, given that unguided iCBT, rather than guided iCBT, was used in this study.

### 4.2. Interpreting Findings against the Literature

Contrary to the findings in this study, an RCT to assess the superiority of the combination strategy of rTMS and bright-light therapy (BLT) over rTMS treatment alone in reducing depressive symptoms in TRD reported significant improvement in the depressive symptoms of participants in the rTMS plus BLT group, as recorded on the 17-item HAMD, compared to the rTMS alone group [71]. The possible explanation for this may be that, unlike unguided iCBT in our study, the BLT could enhance and accelerate the antidepressant effect of rTMS in treating TRD patients by acting as a rapid antidepressant tool involving several pathways through the circadian rhythm regulation and a non-circadian-rhythm-dependent manner.

However, even though this study failed to find an additive value for iCBT to rTMS in regard to the management of TRD symptoms, it does not invalidate the full use and therapeutic efficacy of iCBT in the management of TRD, as there is evidence that supports the use of iCBT for the management of depression and resistant depression [33,44,46,72,73,74,75], with claims of efficacy equivalent to those of CBT delivered by trained personnel [44,72].

A possible reason that may account for the failure of a combination of rTMS and unguided iCBT in our study may be attributed to the differences in the effectiveness of unguided iCBT versus guided iCBT. There is enough evidence in the literature supporting the effectiveness of guided iCBT over unguided iCBT [75,76]. A meta-analysis conducted in this regard found that therapist-guided iCBT demonstrated a greater symptom reduction (d = 0.61) compared to unguided iCBT (d = 0.25) [76]. The reasons for the differences in the forms of iCBT, according to the researchers, were attributed to the added motivation received from the assistance of the therapist and compliance influenced by the guided interventions. In another meta-analysis of 19 RCTs, the researchers again observed the superior effects of guided iCBT (therapist support, d = 0.78; administrative support, d = 0.58) compared to unguided iCBT (d = 0.36) for the management of depression [75]. Thus, unguided iCBT interventions seem to result in more modest outcomes and higher dropout rates compared to therapist-guided iCBT interventions.

Furthermore, geographical differences may also account for the modest outcomes of unguided iCBT delivered through the MoodGYM platform to Canadian patients. Thus, cultural references in the usage of this internet-based intervention platform may be more familiar to individuals living in Australia, where it was developed, compared to North America and Europe—hence, the demonstration of large effect sizes for MoodGYM RCTs conducted in Australia (g = 0.73, 95% CI: 0.19–1.27) compared to the small effect sizes displayed for RCTs conducted in Europe (g = 0.17, 95% CI: 0.04–0.30) [77]. Again, Australia is well-known for its expertise in the development of computer-based psychotherapeutic interventions with excellent infrastructure relating to the administration of these interventions [78]. Therefore, the possibility that the superiority of effectiveness within RCTs conducted in Australia is somehow attributed to the greater acceptance of iCBT for that matter MoodGYM in this population it is noteworthy.

In summary, the present study is highly informative given the fact that it is the first of its kind. However, the study did not find the combined treatment of rTMS + iCBT (unguided) superior over rTMS alone over short-term effects. Hence, we can accept the null hypothesis, as our findings indicated no statistical differences between the two treatment interventions under study in terms of their MDD symptoms, suicidal ideations, subjective MDD symptoms, and health status. Thus, this result does not support the use of combined treatment of rTMS + unguided iCBT for the management of TRD disorders.

### 4.3. Strengths of This Study

The randomization of participants ensured that there was a balanced distribution of different characteristics of participants between the two treatment arms at baseline. Primary outcome assessors were blinded to treatment group allocation by not involving them in discussions about study participants and not granting them access to the secured database which contained the randomization codes. After data collection was completed, all data underwent a blind review to finalize the planned analysis. The blinding of the primary outcome assessors for the primary outcome measures ensured the elimination of bias in outcome measures.

## 5. Limitation

This study has several limitations which need to be considered when interpreting the findings. First, the sample size of study participants who completed both the baseline and 6-week assessments was small. Thus, the small sample size might have impacted the study power, limiting the ability of the study to detect differences in outcome measures between participants in the two treatment arms at discharge. Therefore, our results may not be generalizable to the general population and should be interpreted with caution. Secondly, since the study employed self-guided iCBT, participants were encouraged to conduct the iCBT sessions on their own in the comfort of their homes. While the research team used continuous reminders via text messaging about the iCBT sessions to participants, there was no direct supervision, and, hence, the participants’ adherence to the protocols of this platform, MoodGYM, cannot be guaranteed. However, the adopted analysis was the intention to treat, which follows the initial assigned groups regardless of the actual received treatment. Thirdly, the possible variability in illness duration and concomitant treatments (medication and/or psychotherapy) outside the rTMS clinic being received by patients, as well as the variability in the length of time to be spent by patients in the two arms of the study, could have had some level of confounding effects on the outcomes of our interventions.

## 6. Conclusions

This study failed to demonstrate a significant difference regarding the management of MDD symptoms, subjective MDD, suicidal ideations, and the quality of health between rTMS alone and rTMS plus unguided iCBT on all scales. Many factors may have accounted for the lack of significant differences in our intervention groups. To address these factors, future studies need to investigate the cross-cultural factors that may influence the effectiveness of iCBT interventions in North America if delivered by the MoodGYM program. It is recommended that future studies on the combination of rTMS+ unguided iCBT be run on a large sample size and for a longer term in patients with TRD. This may provide evidence to support the implementation and upscaling of the concomitant application of these interventions in a way that fits the needs of the targeted population.

## Figures and Tables

**Table 1:** Baseline distribution of sociodemographic and clinical characteristics between the two study groups at baseline.

Variables	rTMS	iCBT + rTMS	Total	Chi-Square/*t*-Test	*p*-Value
	*n* = 39	*n* = 39			
**Gender**					
Male	17 (43.6%)	11 (28.2%)	28 (35.9%)		
Female	22 (56.4%)	28 (71.8%)	50 (64.1%)	χ^2^(1) = 2.01	0.24
**Age (Years)**					
≤25	3 (7.7%)	3 (7.7)	6 (7.7%)		
26–40	19 (48.7%)	14 (35.9%)	33 (42.3%)		
>40	17 (43.6%)	22 (56.4%)	39 (50.0%)	1.47 *	0.54
**Educational level**					
Elementary	1 (2.8%)	1 (2.8%)	2 (2.7%)		
High school	8 (22.2%)	10 (26.3%)	18 (24.3%)		
College/University	27 (75.0%)	27 (71.1%)	54 (73.0%)	0.43 *	0.89
**MDD at baseline**					
At most mild depression	18 (54.5%)	16 (43.2%)	34 (48.8%)		
Moderate-to-severe depression	15 (45.5%)	21 (56.8%)	36 (51.4%)		
				χ^2^(1) = 0.89	0.47
**Suicidal ideation at baseline**					
No suicidal ideation	14 (40.0)	7 (18.4)	21 (28.8)		
Present suicidal ideation	21 (60.0)	31 (81.6)	52 (71.2)	χ^2^(1) = 4.14	0.07
**Subjective depression at baseline (QIDS)**					
At most mild depression					
Moderate-to-severe depression	4 (11.8)	3 (8.1)	7 (9.9)		
	30 (88.2)	34 (91.9)	64 (90.1)	χ^2^(1) = 0.27	0.7
**EQ-5D-5L at baseline**					
**Mobility:**					
No problems walking					
Slight problems walking	22 (64.7)	28 (75.7)	50 (70.4)		
Moderate problems walking	8 (23.5)	2 (5.4)	10 (14.1)		
Severe problems walking	3 (8.8)	5 (13.5)	8 (11.3)		
Unable to walk	1 (2.9)	2 (5.4)	3 (4.2)	5.01 *	0.15
**Self-care:**					
No problems washing/dressing					
Slight problems washing/dressing	18 (52.9)	21 (56.8)	39 (54.9)		
Moderate problems washing/dressing	10 (29.4)	5 (13.5)	15 (21.1)		
Severe problems washing/dressing	5 (14.7)	7 (18.9)	12 (16.9)		
Unable to washing/dressing	1 (2.9)	4 (10.8)	5 (7.0)	3.91	0.27
**Usual activities**					
No problems doing usual activities	7 (20.6)	2 (5.4)	9 (12.7)		
Slight problems doing usual activities	7 (20.6)	9 (24.3)	16 (22.5)		
Moderate problems doing usual activities	12 (35.3)	9 (24.3)	21 (29.6)		
Severe problems doing usual activities					
Unable to do usual activities	7 (20.6)	15 (40.5)	22 (31.0)	6.51 *	0.15
	1 (2.9)	2 (5.4)	3 (4.2)		
**Pain/discomfort**					
No pain or discomfort	13 (38.2)	7 (18.9)	20 (28.2)		
Slight pain or discomfort	6 (17.6)	14 (37.8)	20 (28.2)		
Moderate pain or discomfort	10 (29.4)	11 (29.7)	21 (29.6)		
Severe pain or discomfort	4 (11.8)	5 (13.5)	9 (12.7)		
Extreme pain or discomfort	1 (2.9)	0 (0.0)	1 (1.4)	5.95 *	0.17
**Anxiety/depression**					
Not anxious or depressed	1 (2.9)	1 (2.7)	2 (2.8)		
Slightly anxious or depressed	4 (11.8)	0 (0.0)	4 (5.6)		
Moderately anxious or depressed	13 (38.2)	13 (35.1)	26 (36.6)		
Severely anxious or depressed	10 (29.4)	18 (48.6)	28 (39.4)		
Extremely anxious or depressed	6 (17.6)	5 (13.5)	11 (15.5)	6.22 *	0.15
**Total score HAMD at baseline**	*n* = 33	*n* = 37	-		
Mean score (SD)	15.73 (6.03)	17.43 (4.79)		t (68) = 1.32	0.19
**Total score CSSRS baseline**	*n* = 35	*n* = 38	-		
Mean score (SD)	1.57 (1.70)	1.79 (1.44)		t (71) = 0.59	0.56
**Total score QIDS baseline**	*n* = 34	*n* = 37	-		
Mean score (SD)	16.62 (4.59)	17.59 (4.15)		t (69) = 0.94	0.35
**EQ-VAS at baseline mean score (SD)**	*n* = 33	*n* = 37	-		
	47.48 (18.42)	45.27 (18.36)	-	t (68) = 0.50	0.62

* Fisher Exact test was applied.

**Table 2:** Descriptive mean scores of outcome measures and ANCOVA test parameters for the rTMS and rTMS + iCBT groups.

Measure	Descriptive				ANCOVA Parameters		
	Baseline, Mean (SD)		Discharge Means (SD)		F Value (df)	*p*-Value	Partial Eta
	rTMS	rTMS + iCBT	rTMS	rTMS + iCBT			
**HAM-D-17**	15.73 (6.03)	17.43 (4.79)	8.89 (5.83)	9.97 (7.03)	0.15 (1)	0.70	0.003
**CSSRS**	1.57 (1.70)	1.79 (1.44)	0.96 (1.45)	1.03 (1.11)	0.04 (1)	0.85	0.001
**QIDS-16**	16.62 (4.59)	17.59 (4.15)	10.08 (4.36)	11.34 (5.72)	0.26 (1)	0.61	0.005
**EQ-VAS**	65.80 (17.06)	59.76 (21.72)	64.42 (18.13)	60.90 (21.45)	0.46 (1)	0.50	0.009

**Table 3:** Distribution of the prevalence of the clinical characteristics between the two study groups at discharge.

Measures	rTMS	iCBT + rTMS	Total	Chi-Square/Fischer Exact	*p*-Value
	*n* (%)	*n* (%)			
**MDD at discharge**	*n* = 27	*n* = 30			
At most mild depression	24 (88.9)	25 (83.3)	49 (86.0)	*	0.71
Moderate-to-severe depression	3 (11.1)	5 (16.7)	8 (14.0)		
**Suicidal ideation at discharge**	*n* = 28	*n* = 31			
No suicidal ideation	16 (57.1)	12 (38.7)	28 (47.5)	2.01	
Present suicidal ideation	12 (42.9)	19 (61.3)	31 (52.5)		0.2
**Likely depression at discharge (QIDS)**					
At most mild depression	*n* = 26	*n* = 32			
Moderate-to-severe depression	13 (50.0)	19 (59.4)	32 (55.2)		
	13 (50.0)	13 (40.6)	26 (44.8)	0.51	0.6
**EQ-5D-5L at discharge**					
**Mobility:**					
No problems walking	17 (65.4)	23 (74.2)	40 (70.2)		
Slight problems walking	7 (26.9)	5 (16.1)	12 (21.1)		
Moderate problems walking	1 (3.8)	1 (3.2)	2 (3.5)		
Severe problems walking	1 (3.8)	2 (6.5)	3 (5.3)		
Unable to walk	0 (0.0)	0 (0.0)	0 (0.0)	1.48 *	0.81
**Self-care:**					
No problems washing/dressing					
Slight problems washing/dressing	17 (65.4)	19 (61.3)	36 (63.2)		
Moderate problems washing/dressing	7 (26.9)	8 (25.8)	15 (26.3)		
Severe problems washing/dressing	1 (3.8)	3 (9.7)	4 (7.0)		
Unable to washing/dressing	1 (3.8)	1 (3.2)	2 (3.5)	0.98	0.91
**Usual activities**					
No problems doing usual activities	7 (26.9)	4 (12.9)	11 (19.3)		
Slight problems doing usual activities	9 (34.6)	15 (48.4)	24 (42.1)		
Moderate problems doing usual activities	6 (23.1)	6 (19.4)	12 (21.1)		
Severe problems doing usual activities	3 (11.5)	5 (16.1)	8 (14.0)		
Unable to do usual activities	1 (3.8)	1 (3.2)	2 (3.5)	2.66	0.67
**Pain/discomfort**					
No pain or discomfort	12 (46.2)	13 (41.9)	25 (43.9)		
Slight pain or discomfort	7 (26.9)	9 (29.0)	16 (28.1)		
Moderate pain or discomfort	6 (23.1)	6 (19.4)	12 (21.1)		
Severe pain or discomfort	0 (0.0)	2 (6.5)	2 (3.5)		
Extreme pain or discomfort	1 (3.8)	1 (3.2)	2 (3.5)	1.83 *	0.87
**Anxiety/depression**					
Not anxious or depressed	4 (15.4)	4 (12.9)	8 (14.0)		
Slightly anxious or depressed	6 (23.1)	11 (35.5)	17 (29.8)		
Moderately anxious or depressed	10 (38.5)	8 (25.8)	18 (31.6)		0.59
Severely anxious or depressed	5 (19.2)	8 (25.8)	13 (22.8)		
Extremely anxious or depressed	1 (3.8)	0 (0.0)	1 (1.8)	2.95 *	

* Fisher Exact test was applied.

**Table 4:** Comparison of the baseline and 6-week mean scores on the HAMD-17, CSSRS, QIDS, and EQ-VAS scales for study participants who completed both the baseline and sixth-week scales (*n* = 76).

Measure	Responses, *n*	Scores			Mean Difference (95% CI)	*p*-Value	*t* Value	Effect Size (Cohen d)
		Baseline Score, Mean (SD)	Six-Week Score, Mean (SD)	Change from Baseline, %				
**HAMD**	56	16.25 (5.29)	9.45 (6.44)	41.8	6.80 (4.98–8.63)	<0.001	7.46	1.15
**CSSRS**	59	1.69 (1.59)	1.00 (1.27)	40.8	0.69 (0.35–1.04)	<0.001	4.06	0.48
**QIDS**	56	16.79(4.45)	10.88 (5.22)	35.2	5.91 (4.66–7.16)	<0.001	9.45	1.23
**EQ-VAS**	54	47.67 (18.45)	62.56 (19.75)	61.56	14.89 (−19.62)–(−10.16)	<0.001	6.31	0.78

**Table 5:** Comparison of the baseline and 6-week prevalence of major depressive disorder, suicidal ideations, subjective depression, and the quality of health for all study participants.

Condition	Prevalence, *n*/Total Responses (%)		Change in Prevalence (the Sixth Week from Baseline), %	χ^2^ (df)	*p*-Value
	Baseline	Sixth Week			
**MDD clinical diagnosis**	27/56 (48.2)	8/56 (14.3)	−33.9	15.00 (1)	<0.001
**Suicidal ideations**	42/59 (71.2)	31/59 (52.5)	−18.7	4.35 (1)	0.004
**Subjective depression (QIDS-16)**	49/56 (87.5)	26/56 (46.4)	−41.1	21.35 (1)	<0.001
**EQ-5D-5L**					
**Mobility:**					
No problems walking			−3.6		
Slight problems walking	41/55 (74.5)	39/55 (70.9)	10.9		
Moderate problems walking	6/55 (10.9)	12/55 (21.8)	−7.3		
Severe problems walking	5/55 (9.1)	1/55 (1.8)	0	4.72(1)	0.194
Unable to walk	3/55 (5.5)	3/55 (5.5)			
**Self-care:**					
No problems washing/dressing	32/55 (58.2)	35/55 (63.6)			
Slight problems washing/dressing	11/55 (20.0)	14/55 (25.5)	5.4		
Moderate problems washing/dressing	11/55 (20.0)	4/55 (7.3)	5.5		
Severe problems washing/dressing	1/55 (1.8)	2/55 (3.6)	−12.7	4.09 (1)	0.252
Unable to washing/dressing			1.8		
**Usual activities**					
No problems doing usual activities	8/55 (14.5)	11/55(20.0)	5.5		
Slight problems doing usual activities	14/55 (25.5)	23/55 (41.8)	16.3		
Moderate problems doing usual activities	16/55 (29.1)	11/55 (20.0)	−9.1		
Severe problems doing usual activities	15/55 (27.3)	8/55 (14.5)	−12.8	5.72 (1)	0.221
Unable to do usual activities	2/55 (3.6)	2/55 (3.6)	0		
**Pain/discomfort**					
No pain or discomfort	15/55 (27.3)	24/55 (43.6)	16.3		
Slight pain or discomfort	18/55 (32.7)	16/55 (29.1)	-3.6		
Moderate pain or discomfort	16/55 (29.1)	11/55 (20.0)	-9.1		
Severe pain or discomfort	5/55 (9.1)	2/55 (3.6)	-5.5	4.74 (1)	0.315
Extreme pain or discomfort	1/55 (1.8)	2/55 (3.6)	1.8		
**Anxiety/depression**					
Not anxious or depressed	2/55 (3.6)	7/55 (12.7)	9.1		
Slightly anxious or depressed	3/55 (5.5)	16/55 (29.1)	23.6		
Moderately anxious or depressed	18/55 (32.7)	18/55 (32.7)	0		
Severely anxious or depressed	21/55 (38.2	13/55 (23.6)	−14.6	21.89 (1)	<0.001
Extremely anxious or depressed	11/55 (20.0)	1/55 (3.6)	−16.6		

## Data Availability

The raw data supporting the conclusions of this article will be made available by the authors, without undue reservation.
